# The Role of Echocardiography in the Assessment of Epicardial Adipose Tissue: A Systematic Review and Meta-analysis

**DOI:** 10.1007/s13679-026-00734-3

**Published:** 2026-07-23

**Authors:** Barbara Pala, Mariagrazia Piscione, Francesco Cribari, Serena De Mitri, Paola Gualtieri, Antonino De Lorenzo, Luigi Barrea, Marco Alfonso Perrone, Gianluca Iacobellis, Laura Di Renzo

**Affiliations:** 1 PhD School of Applied Medical-surgical Sciences, Tor Vergata University of Rome, Via Montpellier, 1, Rome, 00133 Italy; 2 UOC Cardiologia Ospedale IDI- IRCCS, Via Monti di Creta, 104, Rome, 00167 Italy; 3 ASL 2 Abruzzo, Santissima Annunziata Hospital, Via dei Vestiti, Chieti, 66100 Italy; 4 Section of Food Science, Clinical Nutrition and Pharmaceutical Sciences, Department of Biomedicine and Prevention, Tor Vergata University of Rome, Via Montpellier 1, Rome, 00133 Italy; 5 Dipartimento di Psicologia e Scienze della Salute, Università Telematica Pegaso, Naples, Italy; 6 Division of Cardiology and CardioLab, Department of Clinical Sciences and Translational Medicine, Tor Vergata University of Rome, Via Montpellier,1, Rome, 00133 Italy; 7 Division of Endocrinology, Diabetes and Metabolism, Department of Medicine, University of Miami, Leonard M. Miller School of Medicine, Miami, FL USA

**Keywords:** Epicardial adipose tissue, Trans-thoracic echocardiography, Cardiac computed tomography, Cardiac magnetic resonance, Accuracy

## Abstract

**Background:**

Epicardial adipose tissue (EAT) is a metabolically active visceral fat depot implicated in cardiometabolic and cardiovascular (CV) disease. Although cardiac magnetic resonance (CMR) and cardiac computed tomography (cCT) enable accurate volumetric quantification of EAT, their cost, limited availability, and—particularly for cCT—radiation exposure, restrict their use in preventive and longitudinal settings. Transthoracic echocardiography (TTE) is widely accessible and radiation-free, but its validity as a surrogate of volumetric EAT assessment and its broader clinical role remain incompletely defined.

**Objectives:**

To systematically synthesize disease-specific evidence linking TTE-derived EAT thickness with major CV phenotypes and to quantitatively assess its association with volumetric EAT measured by CMR or cCT.

**Methods:**

A systematic search of PubMed and PubMed Central (January 2000–December 2025) identified adult studies evaluating associations between TTE-derived EAT thickness and coronary artery disease (CAD), atrial fibrillation (AF), or heart failure with preserved ejection fraction (HFpEF), and correlations between TTE-derived EAT thickness and CMR- or cCT-derived EAT volume. Correlation coefficients were pooled using a random-effects model after Fisher’s z-transformation. An exploratory meta-analysis assessed associations with major adverse cardiovascular events (MACE).

**Results:**

Seventeen disease-specific studies consistently demonstrated associations between increased TTE-derived EAT thickness and CAD severity, AF burden and recurrence, and adverse HFpEF phenotypes. Five validation studies were included; four comparing TTE with CMR were pooled, yielding a moderate-to-strong correlation (*r* = 0.77, 95% CI 0.65–0.93; *p* < 0.01; I² = 92.9%). Several studies reported associations between TTE- EAT thickness and cCT-derived EAT parameters. However, only one cCT study met our predefined criteria for inclusion in the quantitative validation analysis, whereas the remaining cCT studies were retained in the qualitative synthesis because of substantial methodological heterogeneity in cCT acquisition, segmentation approaches, and outcome definitions. Exploratory prognostic analysis suggested a directional association between increased TTE-derived EAT thickness and MACE.

**Conclusions:**

TTE-derived EAT thickness correlates with volumetric EAT and is consistently associated with major CV phenotypes. Standardization and prospective outcome-driven validation are required before routine clinical implementation.

**Graphical Abstract:**

**Figure:**
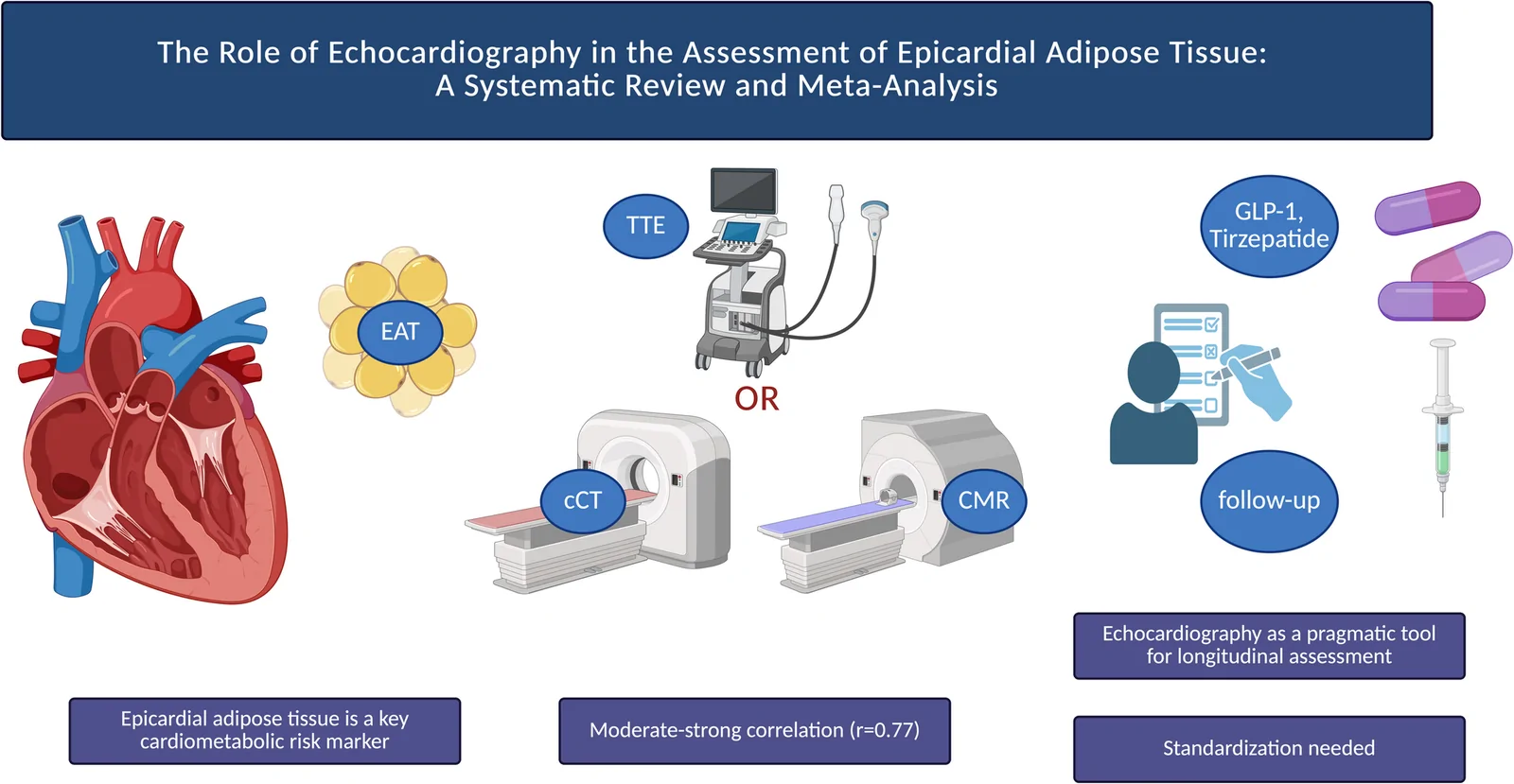
This graphical abstract summarizes the relationship between transthoracic echocardiography–derived epicardial adipose tissue (EAT) thickness and volumetric EAT assessment obtained by cardiac computed tomography or cardiac magnetic resonance imaging. While cross-sectional imaging techniques provide accurate three-dimensional quantification of EAT, their use is limited in preventive and longitudinal settings. Echocardiography offers a pragmatic, radiation-free alternative that allows rapid assessment of EAT. The figure highlights the significant association between one-dimensional echocardiographic measurements and volumetric EAT, as well as the clinical implications for large-scale screening and follow-up. Methodological heterogeneity and the need for standardization are emphasized as key limitations for clinical translation.

## Introduction

### Epicardial Adipose Tissue: Definition and Biological Role

Epicardial adipose tissue (EAT) is a specific visceral adipose tissue depot located between the myocardium and the visceral layer of the pericardium, in direct contact with the coronary arteries and myocardium [1]. This anatomical feature allows direct contact between EAT, the underlying myocardium and coronary vasculature, facilitating close paracrine and vasocrine interactions [1].

A relevant component of EAT is represented by pericoronary adipose tissue (PCAT), defined as the adipose tissue located within a radial distance from the outer coronary vessel wall equal to the diameter of the adjacent coronary artery [2]. In large epicardial vessels, PCAT is contiguous with the adventitial layer, whereas in small vessels and microvessels, perivascular adipose tissue adipocytes constitute an integral component of the vascular wall itself [2].

It is essential to distinguish EAT from other cardiac-related fat depots [2]. Pericardial adipose tissue (PAT) is defined as adipose tissue located between the visceral and parietal layers of the pericardium, while paracardial adipose tissue refers to fat located externally to the parietal pericardium [2]. Unlike EAT, these depots are anatomically separated from the myocardium and coronary arteries and lack direct biological interaction with cardiac structures [3].

In the normal heart, EAT covers approximately 80% of the cardiac surface [3]. Its distribution is heterogeneous, being predominantly concentrated along the atrioventricular (AV) and interventricular (IV) grooves and around the epicardial coronary arteries [3]. Smaller quantities of EAT are found surrounding the atria, over the free wall of the right ventricle, and at the apex of the left ventricle [3, 4].

From a histological perspective, EAT originates from the splanchnopleuric mesoderm and is composed of adipocytes that are significantly smaller than those found in subcutaneous and intra-abdominal fat depots [5]. During early life, EAT exhibits a predominantly brown adipose tissue phenotype, while in middle age it acquires features of so-called “beige” fat, characterized by a combination of white and brown adipocytes. In addition to adipocytes, EAT contains inflammatory, stromal, immune and nervous cells [5].

Traditionally, EAT has been regarded as a protective structure for the heart since it supports the coronary arteries against torsional stress induced by arterial pulsatility and cardiac contraction [5]. Moreover, it provides an immunologically active barrier against pathogens by surrounding cardiac structures with immune surveillance [5]. Due to its brown and beige adipocyte components, EAT contributes to thermogenesis and may protect the heart from hypothermia [5].

Beyond these metabolic and mechanical roles, EAT is now recognized as an active endocrine organ with a distinct secretory profile [6]. It releases a wide array of bioactive molecules, including cytokines, growth factors, and vasoactive hormones—collectively referred to as adipokines [6]. These mediators exert effects on adjacent myocardium and coronary arteries, as well as through the local microcirculation [6]. In physiological conditions, EAT-derived adipokines promote vasodilation and exert anti-inflammatory, anti-fibrotic, and anti-oxidative effects, contributing to cardiovascular (CV) homeostasis [6].

### Clinical Relevance of EAT

While in healthy population, EAT plays a central role ranging from energy storage and thermal insulation to mechanical protection, excess EAT has been constantly associated with coronary artery disease (CAD), particularly in obese and insulin-resistant subjects [7]. The relationship between EAT and CAD is thought to be mediated both by chronic low-grade systemic inflammation driven by adipokine dysregulation and by direct local effects of EAT on the vascular wall [8]. While the classical “inside-out” hypothesis attributes atherosclerosis to primary endothelial injury and subsequent inflammatory cell infiltration of the intima, recognition of EAT as an active inflammatory organ has led to the development of the complementary “outside-to-inside” theory [9]. According to this model, vascular inflammation may originate in the surrounding EAT and propagate inward toward the vessel wall [9].

Under pathological conditions such as excess caloric intake and insulin resistance, EAT undergoes phenotypic remodelling which is characterized by adipocyte hypertrophy, impaired triglyceride storage, enhanced lipolysis with increased release of free fatty acids, and activation of inflammatory pathways [10]. Dysfunctional EAT exhibits a marked shift in its secretory profile, with amplified production of pro-inflammatory adipokines including leptin, resistin, monocyte chemoattractant protein-1, and interleukin-8 (IL-8) [10].

Among EAT sub-compartments, PCAT may exert a particularly direct role in coronary atherosclerosis due to its intimate anatomical proximity to the coronary arterial wall [11]. This close relationship enables endothelial dysfunction, reduced nitric oxide bioavailability, a pro-thrombotic state, and vascular smooth muscle cell proliferation [12].

Beyond CAD, recent evidence indicates that EAT is implicated in a broad spectrum of CV and systemic conditions [13]. EAT volume and thickness has been consistently correlated with atrial fibrillation (AF) prevalence, burden, and recurrence after catheter ablation [13]. As a matter of fact, EAT-derived pro-inflammatory cytokines and reactive oxygen species promote atrial fibrosis and conduction heterogeneity [13]. Moreover, EAT has been shown to infiltrate the atrial myocardium and ganglionated plexi, contributing to autonomic dysregulation so that it creates a substrate conducive to AF initiation and maintenance [14].

Beyond AF, EAT is associated with heart failure with preserved ejection fraction (HFpEF) [15]. The expansion of EAT exert an external mechanical constraint on the heart, limiting diastolic filling and contributing to increased intracardiac pressures [15]. Beyond this passive effect, EAT releases pro-fibrotic mediators, which can diffuse directly into the adjacent myocardium due to the absence of a fascial barrier [16]. This paracrine signaling promotes increased ventricular stiffness and impaired relaxation [17]. In addition, EAT is associated with enhanced lipolytic activity contributing to lipotoxicity, mitochondrial dysfunction and endothelial impairment within the coronary microcirculation [18].

In addition, EAT has been investigated in the context of autoimmune and systemic inflammatory diseases [19]. Patients with chronic inflammatory disorders often exhibit disproportionate expansion and inflammation of visceral adipose tissue (VAT), including EAT, despite relatively modest levels of generalized obesity [20]. In these conditions, EAT may act as a local amplifier of systemic inflammation, contributing to accelerated atherosclerosis, myocardial involvement, and arrhythmic risk [17]. The shared inflammatory pathways linking autoimmune disease activity, EAT dysfunction, and CV involvement support the idea of EAT as an integrative marker of inflammatory CV risk [17].

EAT was also evaluated in relation to body composition parameters. An increase in EAT may occur in presence of BMI elevation, potentially contributing to an increased cardiovascular risk [21, 22]. These considerations underscore the need for effective prevention strategies at multiple levels—including primary prevention, early interventions in younger populations, and secondary prevention—within a global cardiovascular risk reduction framework.

Collectively, these observations make EAT considered a central mediator of metabolic, inflammatory, hormonal, and electrophysiological pathways [18]. This broad clinical relevance provides a strong rationale for accurate, reproducible, and widely applicable non-invasive imaging techniques aimed at quantifying EAT and characterizing its biological activity across different disease states.

### Imaging Assessment of EAT

Cardiac magnetic resonance (CMR), cardiac computed tomography (cCT) and trans-thoracic echocardiography (TTE) can be used to assess EAT.

CMR is considered the reference imaging modality for the assessment of total body fat [20]. Moreover, owing to its excellent soft-tissue contrast, CMR allows clear visualization of both the visceral and parietal pericardium, enabling accurate delineation and volumetric quantification of EAT [20]. Furthermore, CMR does not involve ionizing radiation or the use of contrast agents [20]. However, its clinical application is limited by relatively high costs, reduced availability, longer acquisition times, and contraindications in patients with claustrophobia or non-MRI-compatible implanted devices [23].

Several CMR sequences can be used for EAT assessment. Fast spin-echo T1-weighted black-blood sequences have demonstrated high reliability for EAT quantification in research settings, although they are not routinely acquired in standard clinical CMR protocols [24]. In contrast, steady-state free precession (SSFP) cine images—commonly used for the assessment of ventricular volumes and function—provide an accurate and reproducible evaluation of pericardial and epicardial fat and are therefore more widely applicable in clinical practice [25, 26]. Leo et al. (2019), shows as CMR with SSFP sequences, allows a detailed visualization of EAT especially beyond the epicardial surface, occupying anatomical spaces that are minimal or virtual separating cardiac structures which lie in close apposition (interatrial groove, AV septum, inferior pyramidal space, left lateral ridge, AV grooves, and transverse pericardial sinus) [27].

EAT volume is typically quantified by manually contouring the epicardial fat area on contiguous short-axis slices at end-diastole; the measured areas are multiplied by slice thickness and summed across all slices to obtain total EAT volume [28]. Nevertheless, when assessing EAT the anatomical location and the measurement approach are critical [28]. Accordingly, simpler and more feasible techniques should be preferred whenever they do not compromise measurement accuracy, reproducibility, or sensitivity. This consideration provides the rationale for comparing multi-slice and single-slice assessment strategies [29].

The volumetric approach (multi-slice measurements), analogous to Simpson’s method for the calculation of left ventricular ejection fraction, is considered the reference standard, as it enables a comprehensive evaluation of total EAT burden [29]. However, it is labour- intensive since it requires manual delineation of EAT contours across all short-axis slices [29]. In contrast, single-slice assessment relies on representative views, typically obtained at the AV groove or in the four-chamber view, offering greater technical simplicity but limited sensitivity [29].

As highlighted by Requena-Ibanez et al., the heterogeneous anatomical distribution of EAT supports the preferential use of three-dimensional volumetric assessment (multi-slice measurements) in specific clinical settings where single-slice measurements may fail to capture subtle but relevant variations, particularly when monitoring temporal changes or treatment-related effects [30].

More recently, fully automated approaches for EAT quantification from non-contrast CMR images have been developed, showing promising accuracy and reproducibility and potentially facilitating broader clinical and research use [31]. Beyond volumetric assessment, advanced CMR techniques such as proton magnetic resonance spectroscopy (¹H-MRS) enable non-invasive quantification of intracellular lipid accumulation within the myocardium and liver, providing a direct evaluation of ectopic fat deposition [32]. Unlike conventional imaging, ¹H-MRS measures metabolic lipid content rather than macroscopic fat thickness, thereby offering insight into tissue-specific lipotoxicity and its potential impact on myocardial structure and function [32].

Due to its high spatial resolution and three-dimensional visualization of cardiac structures, cCT is currently considered one of the preferred imaging modalities for the assessment of EAT [33]. Using non-contrast cardiac CT (NCCT), EAT can be reliably identified as adipose tissue with attenuation values ranging from − 190 to − 30 Hounsfield Units (HU), with fat voxels located within the visceral pericardium classified as epicardial fat and those within the inner thoracic cavity defined as thoracic fat [34]. Volumetric quantification of EAT is commonly performed using semi-automated approaches, in which pericardial contours are delineated at predefined intervals from the level of the pulmonary trunk to the cardiac apex, and dedicated software calculates total EAT volume by summing fat areas across contiguous slices while accounting for slice thickness and interslice gaps [35]. Using this methodology, threshold values of EAT volume in the range of approximately 113–120 cm³ have been proposed as optimal predictors of future CV events [36]. Given the time-consuming nature of conventional semi-automated analysis, fully automated software solutions have recently been developed [36].

In contrast to total EAT, the assessment of PCAT requires precise visualization of the coronary artery lumen and vessel wall in order to define the spatial relationship between the vessel and the surrounding fat [37]. For this reason, contrast-enhanced coronary CT angiography (CCTA) is essential for PCAT analysis, as non-contrast imaging does not allow reliable delineation of the coronary tree or accurate localization of perivascular adipose tissue [37, 38]. In the setting of vascular inflammation, pro-inflammatory cytokines released from the coronary wall diffuse into the adjacent PCAT, inhibiting adipocyte differentiation and altering adipocyte maturation [38–40]. These processes lead to smaller adipocytes with reduced intracellular lipid content, increased aqueous fraction and extracellular oedema, resulting in higher CT attenuation values of PCAT [41]. This phenomenon can be quantified by measuring the mean attenuation of fat-containing voxels surrounding the coronary arteries [41].

On this biological and imaging basis, Antonopoulos et al. have demonstrated that physiologically normal PCAT, composed of large, lipid-rich adipocytes, exhibits low attenuation values approaching − 190 HU, whereas inflamed PCAT characterized by smaller, lipid-poor adipocytes shows progressively higher attenuation values, approaching − 30 HU [42]. These observations have led to the introduction of the fat attenuation index (FAI), defined as the mean CT attenuation of PCAT measured within a radial distance from the outer coronary artery wall equal to the diameter of the adjacent vessel [43]. Beyond its role in global CV risk stratification, PCAT attenuation has emerged as a plaque-specific imaging biomarker of coronary inflammation and a potential precursor of culprit lesion development [44].

While CMR and cCT provide accurate volumetric quantification, their cost, limited availability and, for cCT, radiation exposure restrict their applicability in large-scale screening and longitudinal follow-up.

### Why Echocardiography?

The pioneering work of Iacobellis and colleagues first introduces and validates the TTE measurement of EAT, establishing it as a novel, non-invasive surrogate marker of VAT and laying the conceptual and methodological foundation for subsequent research in cardiometabolic imaging [45, 46].

As a matter of fact, TTE represents a promising and impactful tool for the assessment of EAT in routine clinical practice [46]. Its wide availability, low cost, absence of ionizing radiation, and feasibility at the bedside make TTE suited for repeated assessment, longitudinal follow-up, and integration into standard CV evaluation [45, 47]. If reliably standardized, TTE- EAT measurement could enable repeated, real-world assessment of visceral cardiac adiposity and provide information in populations at high cardiometabolic risk, including patients with obesity, type 2 diabetes mellitus (DMT2), and HF [45]. Consistent with this concept, across a series of investigations, Iacobellis and colleagues have proposed threshold values of TTE-EAT thickness to identify individuals at increased cardiometabolic risk [45]. In a large Caucasian cohort, receiver operating characteristic (ROC) analysis suggested that EAT values ≥ 9.5 mm in men and ≥ 7.5 mm in women optimally discriminate subjects with metabolic syndrome and excess VAT, supporting their use as potential high-risk thresholds [47]. Earlier studies had already shown that individuals with visceral obesity exhibited EAT thickness values around 9–10 mm in men and 7–8 mm in women, compared with approximately 3–4 mm in metabolically healthy subjects [48]. Importantly, the clinical relevance of these proposed thresholds was later supported in a pragmatic study of 142 well-controlled, asymptomatic DMT2 patients on metformin monotherapy, in whom mean EAT thickness (8.3 ± 2.3 mm in women and 9.4 ± 2.4 mm in men) exceeded previously proposed high-risk values [49]. In multivariable analysis, EAT thickness was the only independent predictor of incident CAD at 1-year follow-up, outperforming traditional risk markers such as BMI, age, blood pressure, and DMT2 duration [49]. Although these cut-offs were not derived from large prospective outcome-driven trials, they represent one of the earliest attempts to translate EAT measurement into clinical CV risk categories [45–49].

Despite these advantages, TTE has not yet been established as a reference modality for EAT quantification [49]. The main limitations relate to substantial methodological heterogeneity across studies, including variability in imaging views, anatomical landmarks, timing within the cardiac cycle, and measurement techniques [49]. However, such variability should not be interpreted solely as a methodological weakness [46]. Rather, it likely reflects the intrinsic biological variability of VAT distribution across sex, age, and ethnic groups [2]. Indeed, EAT is a dynamic visceral fat depot influenced by hormonal, metabolic, and genetic determinants, and differences in measurement approaches may partly capture population-specific phenotypic expression of VAT [2]. Therefore, while standardization remains essential for comparability and prognostic validation, inter-study variability may also underscore the need for sex- and ethnicity-adjusted reference ranges rather than a single universal threshold [46].

From a clinical perspective, EAT thickness values around 5 mm are often considered within the normal range in several TTE studies, whereas progressively higher measurements appear to reflect a pathologic expansion of visceral cardiac adiposity [49–51]. Accordingly, EAT thickness > 7–9 mm is generally interpreted as indicative of a high-risk phenotype rather than a strict diagnostic cut-off, particularly in the absence of standardized acquisition protocols and universally validated thresholds [49–51].

In addition, in the TTE setting, careful distinction between EAT and pericardial structures is essential, as misclassification may occur particularly in the presence of suboptimal acoustic windows or pericardial abnormalities, which carry different diagnostic and clinical implications [52]. In addition, it can be stated that he main limitation of TTE assessment is its inherent operator dependency, as image acquisition and measurement accuracy are highly influenced by the examiner’s experience and technical expertise [2].

In the available literature, three main TTE approaches have been described for the assessment of EAT [53].

The first and most reported approach involves measurement from the parasternal long (PLAX) and short axis (PSAX) view [54]. In PLAX, EAT thickness is measured perpendicularly to the free wall of the right ventricle at end-systole, using the point perpendicular to the aortic annulus as the anatomical reference. The reported value is typically calculated as the average of three consecutive cardiac cycles acquired at end-systole [54].

The PSAX measurement consists of calculating EAT thickness from the mid-ventricular short axis view. In this setting, EAT is assessed on the right ventricular free wall, perpendicular to the IV septum, at the mid-chordal level, with the tips of the papillary muscles serving as anatomical landmarks [54].

A second TTE technique employs a high-frequency linear probe and focuses on the anterior IV groove [55]. In this approach, EAT thickness is measured at end-systole following visualization of the distal left anterior descending (LAD) coronary artery. After obtaining a modified three-chamber view and further probe rotation to align the LAD longitudinally, EAT thickness is quantified as the distance between the outer myocardial wall and the visceral layer of the pericardium, measured perpendicular to the pericardial surface. This method has been proposed to improve spatial resolution and local assessment of epicardial fat adjacent to the coronary arteries [55].

Finally, a third method involves measurement at the level of the fold of Rindfleisch, just to the right of the aortic annular plane [56]. In this parasternal long-axis view, EAT is visualized between the free wall of the right ventricle and the anterior surface of the ascending aorta, where epicardial fat accumulation is often maximal [56].

As already said, this marked heterogeneity across studies has important implications for result comparability and clinical interpretation [57, 58]. Consequently, reference ranges across different populations are still lacking [54].

These unresolved issues have limited the translation of TTE- EAT assessment from research settings into routine clinical decision-making. However, they also highlight the need for a systematic evaluation of the existing evidence [32]. In this context, a comprehensive synthesis of the literature is required to clarify the principal areas of clinical application of EAT and the strength of the association between TTE-EAT measurements and established imaging modalities, to identify sources of heterogeneity, and to assess whether TTE can provide a reliable and clinically meaningful estimate of epicardial fat burden [59]. This systematic review and meta-analysis therefore aims to critically appraise the role of TTE in EAT assessment and to define its potential position also within the multimodality imaging framework [59].

### Rationale and Assessment of the Study

The primary objective of this systematic review and meta-analysis is to comprehensively evaluate the clinical and imaging evidence regarding TTE assessment of EAT. We first aim to systematically synthesize disease-specific evidence examining the association between TTE–derived EAT thickness and major CV phenotypes, including CAD, AF, and HFpEF.

We further aim to quantify the strength and consistency of the association between one-dimensional TTE-derived EAT thickness and EAT volume assessed by CMR or cCT.

Secondary objectives include exploring potential sources of heterogeneity related to imaging modality, anatomical measurement site, and study population characteristics, as well as performing an exploratory meta-analytic evaluation of the association between TTE-derived EAT thickness and adverse CV outcomes.

## Methods

### Search Strategy and Study Selection

A systematic literature search is conducted using PubMed and PubMed Central to identify relevant studies published between January 2000 and December 2025. The search strategy includes combinations of keywords and Medical Subject Headings (MeSH) related to EAT, TTE, cCT, CMR, EAT and CV outcomes, EAT measurement in patients with CAD, AF, and HFpEF.

Reference lists of all eligible studies are manually screened to identify additional relevant publications. Study selection is performed according to predefined inclusion and exclusion criteria. The review protocol was registered in the International Prospective Register of Systematic Reviews (PROSPERO; Registration No. XXX).

### Eligibility Criteria

Studies are eligible if they:

1. include adult patients (≥ 18 years);
2. quantify EAT thickness by TTE;
3. report quantitative measures of association between modalities.

Paediatric studies, case reports, editorials, and studies not clearly distinguishing epicardial from pericardial fat are excluded. Two reviewers independently screen titles, abstracts, and full texts, with disagreements resolved by consensus.

### Data Extraction

The following data are extracted from each eligible study: study design, population characteristics, and detailed TTE acquisition protocol, including imaging view, anatomical measurement site, cardiac phase, and number of cardiac cycles averaged. For disease-specific clinical studies included in the qualitative synthesis, key study characteristics, measurement protocols, and principal findings are systematically summarized in structured tables. Quantitative pooling is not performed for these analyses due to substantial methodological heterogeneity across studies.

Information regarding cCT or CMR methodology is also collected when applicable.

For validation studies, extracted metrics include correlation coefficients describing the association between TTE- EAT thickness and cCT- or CMR-derived measurements.

For studies reporting longitudinal outcomes, data on CV events, follow-up duration, effect estimates, and corresponding confidence intervals are additionally extracted.

### Outcomes

The primary objective of this study is twofold. First, to systematically synthesize disease-specific clinical evidence examining the association between TTE–derived EAT thickness and major CV phenotypes.

Second, to quantitatively assess the strength of the association between one-dimensional TTE-derived EAT thickness and EAT volume measured by other methods.

A secondary exploratory outcome is the pooled effect estimate describing the association between increased TTE-derived EAT thickness and adverse CV events in studies reporting longitudinal follow-up data.

### Data Synthesis and Statistical Analysis

Quantitative synthesis is performed when at least two studies reported comparable measures of association between TTE–EAT thickness and EAT volume assessed by CMR or cCT. Reported correlation coefficients are transformed using Fisher’s z-transformation prior to pooling and back-transformed for presentation. A random-effects model is applied to account for between-study variability.

Statistical heterogeneity is assessed using the I² statistic, with values of approximately 25%, 50%, and 75% considered indicative of low, moderate, and high heterogeneity, respectively.

The methodological quality of the included studies is independently assessed by two reviewers using the Quality Assessment of Diagnostic Accuracy Studies-2 (QUADAS-2) tool. This tool evaluates four key domains: patient selection, index test, reference standard, and flow and timing. Disagreements in quality assessment are resolved through discussion or consultation with a second reviewer. No quantitative pooling is performed for disease-specific clinical studies (CAD, AF, and HFpEF) due to substantial methodological heterogeneity in study design, patient populations, and measurement protocols; these data are synthesized qualitatively in structured tables.

### Reporting Standards

This systematic review and meta-analysis are conducted and reported in accordance with the Preferred Reporting Items for Systematic Reviews and Meta-Analyses (PRISMA) guidelines [59].

### Exploratory Prognostic Meta-analysis

As an exploratory analysis, we performed a random-effects meta-analysis to evaluate the association between increased TTE-derived EAT thickness and major adverse cardiovascular events (MACE). Studies reporting time-to-event outcomes as hazard ratios (HRs) were pooled using the generic inverse-variance method after logarithmic transformation of effect estimates and corresponding confidence intervals.

**Fig. 1:**
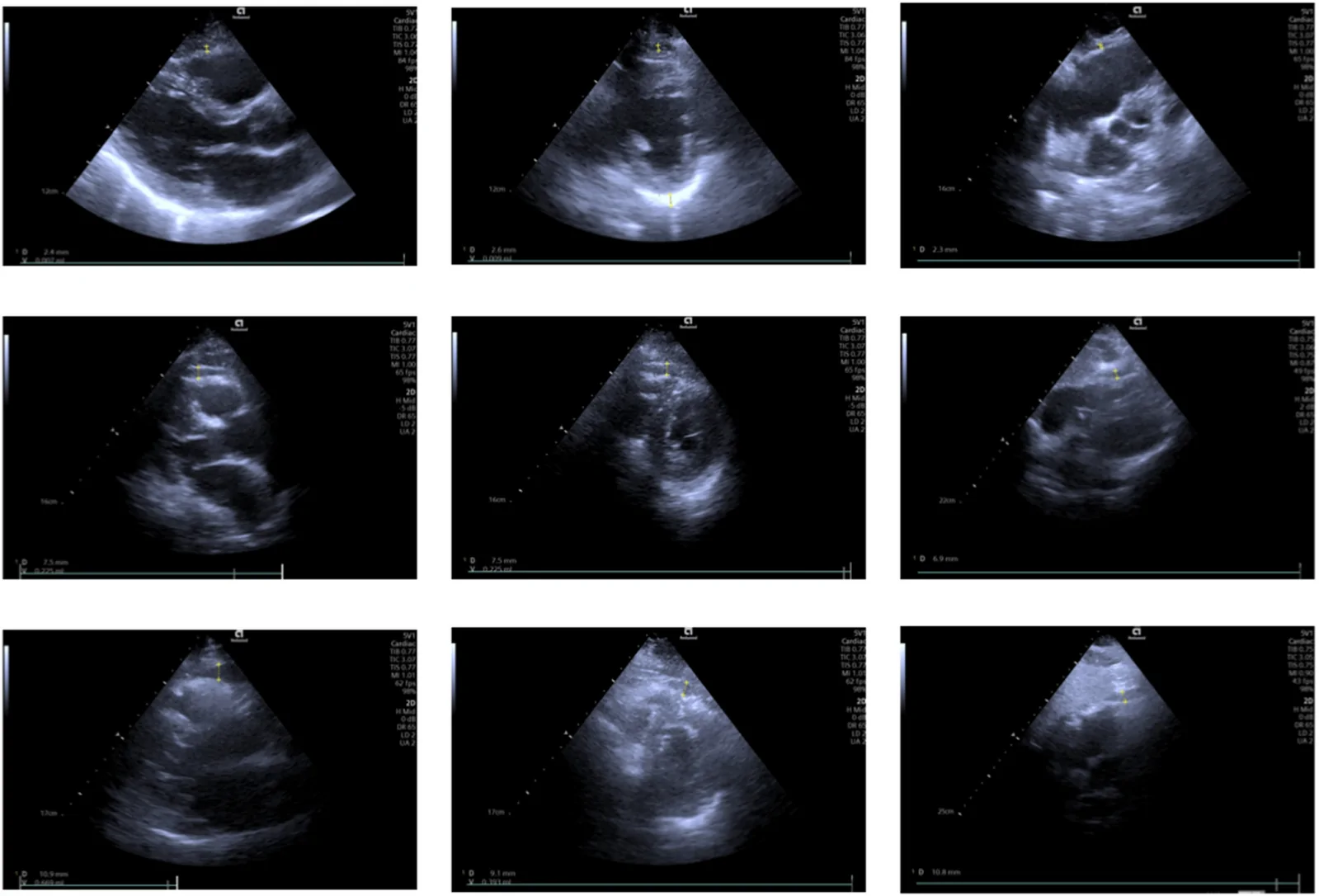
(**A**) Epicardial adipose tissue assessment in parasternal long axis (linear assessment), (**B**) Epicardial adipose tissue assessment in parasternal short axis. (**C**) Epicardial adipose tissue assessment

Given methodological heterogeneity and differences in outcome reporting, the primary prognostic analysis was restricted to studies reporting HR-based estimates derived from dichotomized or continuous TTE-EAT measurements. A predefined sensitivity analysis additionally included one study reporting relative risks (RRs), which were log-transformed and treated as approximations of HRs due to the relatively low event rates and comparable longitudinal framework. For the prognostic meta-analysis, only multivariable-adjusted HRs were extracted and pooled whenever available (Fig. 1).

**Fig. 2:**
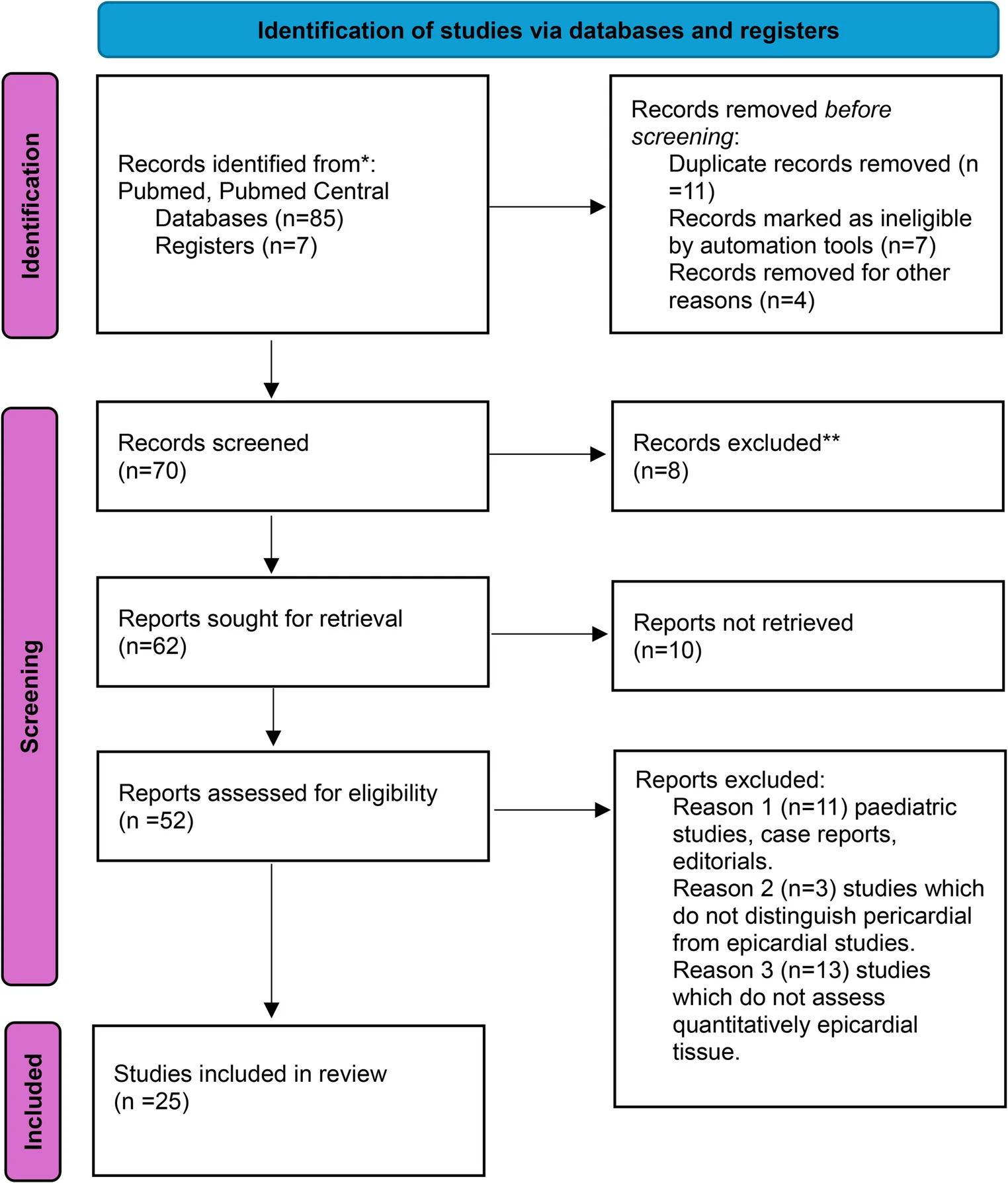
Prisma flow diagram of study selection

Owing to the limited number of available studies and the exploratory nature of the analysis, pooled prognostic estimates were interpreted as hypothesis-generating rather than definitive evidence of prognostic association. **(**Fig. 2**)**.

## Results

### Study Selection

The systematic literature search identifies 92 records through database searching. After removal of duplicates, 70 records are screened by title and abstract, and 45 articles are excluded for not meeting the predefined inclusion criteria.

25 full-text articles are assessed for eligibility. 17 disease-specific clinical studies evaluating the association between TTE-derived EAT thickness and CV phenotypes (CAD, AF, and HFpEF) are identified and included in the qualitative synthesis, not including specific studies analysing specific, dedicated population.

In parallel, five studies meet the criteria for inclusion in the validation meta-analysis and are included in both the qualitative and quantitative synthesis.

The overall study selection process is summarized in the PRISMA flow diagram [59] **(**Fig. 2**)**.

A focused systematic search is additionally conducted to identify studies reporting CV outcomes associated with TTE-derived EAT thickness. The selection process for prognostic analyses is presented in a dedicated PRISMA flow diagram (Fig. 2**)**.

### Study Characteristics

The included studies comprise adult populations with heterogeneous clinical profiles, ranging from apparently healthy individuals to patients with high CV risk or established CV disease. Sample sizes vary substantially across studies, reflecting differences in study design and target populations **(**Tables 1 and 2**).**

**Table 1:** (A-C) Summary of studies describing the evaluation of TTE-EAT in CAD (A), FA (B) and HFpEF (C)

A.CAD					
First Author	Year	Population		EAT Measurement	Main Finding
Jeong et al. [55]	2007	203 patients with suspected CAD		PLAX, end-diastole	EAT is associated with severity of CAD (measured with Gensini’s score at the coronary angiography), OR 10.53, *p* = 0.0004
Ahn et al. [56]	2008	527 patients undergoing their first coronary angiography		PLAX and PSAX, end diastole	EAT is thicker in patients with CAD than in those without CAD (4.0 vs. 1.5, *p* < 0.0001)
Eroglu et al. [57]	2009	150 patients (100 patients with CAD and 50 patients with normal coronary arteries)		PLAX and PSAX, end-diastole	EAT thickness is significantly higher in patients with CAD in comparison to those with normal coronary arteries (6.9 ±1.5 mm vs. 4.4± 0.8 mm; *P* < 0.001).
Park et al. [58]	2010	643 patients who underwent TTE and angiography CAD		PLAX, end-diastole	EAT thickness is correlated with CAD severity especially in patients with BMI < 27 kg/m^2^ (especially in Asian population).
Albuquerque et al. [59]	2012	192 patients		PLAX, end- systole	EAT does not predict for MACE and is poorly associated with measures of obesity in patients with CAD.
Wang et al. [60]	2014	373 patients		PLAX	EAT has a positive correlation with SYNTAX score and is an independent predictor for major in-hospital events.
Zencirci et al. [61]	2014	114 patients with STEMI		PLAX and PSAX	EAT predicted no-reflow phenomenon (3.9 ± 1.7 vs. 5.4 ± 2, *p* = 0.001).
Tanindi et al., [62]	2015	Suspected CAD		PLAX and PSAX	EAT is associated with AMI (cut-off is of 7.8 mm).
Erkan et al. [63]	2016	183 patients with Angina and AMI		PLAX, end-systole	Strong correlation with Gensini & Syntax; cut-off predicted critical CAD.
Morales- Portano et al. [64]	2018	107 patients		PLAX, end- systole	EAT shows higher prediction of MACE than other markers.
B. AF					
First Author	Year	N	Population	Measurement	Main Findings
Chao et al. [65]	2013	283 patients, 227 with paroxysmal AF and 56 with non paroxysmal AF	AF ablation	PLAX	EAT predicts AF recurrence after AF ablation. At cut off of 6 and 6.9 could predict AF recurrences. There is significant correlation between TTE and CT values (*r* = 0.852, *p* < 0.0001)
Iacobellis et al., [66]	2014	84 patients with permanent or paroxysmal AF	Paroxysmal and permanent AF	PLAX, end systole	EAT measured with TTE is significantly higher in patients with chronic AF when compared with patients with paroxysmal AF.
Chu et al., [67]	2016	190 patients with persistent AF	Persistent AF and CV events	PLAX, end diastole	EAT independently predicts CV events
Gunturk et al. [68]	2020	124 patients studied to undergo CABG	CABG patients	PLAX	EAT independently predicts post-operative AF (OR 4.47, 95% CI 3.07–5.87, *p* = 0.001)
C. HFpEF					
First Author	Year	Population		EAT Measurement	Main Finding
Topuz et al. [69]	2017	85 patients with stress test + for CAD and 82 patients without CAD		PLAX (end-diastole)	EAT independently associated with LV diastolic dysfunction
Koepp et al. [70]	2020	169 obese patients with HFpEF		PLAX	EAT ≥ 9 mm associated with higher filling pressures, greater ventricular interdependence, worse pulmonary hypertension, and 20% lower peak VO₂; identifies more severe obese HFpEF phenotype
Gorter et al. [71]	2020	75 HFpEF patients (36% obese) undergoing invasive hemodynamics		PLAX and PSAX RV free wall; end-systole;	EAT associated with elevated filling pressures, reduced exercise capacity, no association with LV filling pressures.

*Abbreviations: *AMI* acute myocardial infarction, *BMI* body mass index, *CABG*: coronary artery bypass grafting, *CAD* coronary artery disease, *CT *computed tomography, *CV* cardiovascular, *EAT* epicardial adipose tissue, *HFpEF* heart failure with preserved ejection fraction, *LV* left ventricle, *MACE* major adverse coronary events, *PLAX* parasternal long axis, *PSAX* parasternal short axis, *RV* right ventricle, *STEMI* ST elevation myocardial infarction, *TTE *trans-thoracic echocardiography

**Table 2:** Summary of studies comparing TTE assessment of EAT with cCT and CMR

Author (year)	Study population	Modalities compared	EAT metric	Echo assessment	CMR assessment	Key results	References
Iacobellis et al., 2003	60 healthy subjects+ 20 obese subjects (validation group)	TTE vs. CMR	TTE EAT thickness vs. CMR EAT volume	PLAX and PSAX views; >10 cardiac cycles; end systole	1.5 T CMR; T1-weighted sequences; 10-mm slice thickness with 1-mm interslice gap	Strong correlation between echocardiographic EAT thickness and CMR-derived EAT volume.	[54]
Iacobellis et al., 2003	72 adults (BMI 22–47 kg/m^2)^	TTE vs. CMR	TTE EAT thickness vs. CMR EAT volume	PLAX and PSAX mean of maximal values	1.5 T CMR; T1-weighted sequences; 10-mm slice thickness with 1-mm interslice gap	TTE-EAT strongly correlates with MRI EAT and abdominal VAT (*r*=0.84–0.91).	[48]
Malavazos et al., 2010	20 patients referred for cardiometabolic risk assessment	TTE vs. CMR vs. H-^1^MR spectroscopy	TTE EAT thickness vs. CMR EAT volume	PLAX and PSAX; 3 cardiac cycles; end systole	1.5 T CMR; SSFP bright-blood sequences; 7-mm slice thickness; no interslice gap	Excellent correlation between TTE and CMR EAT (*r* = 0.90, *p* < 0.01).	[32]
Sicari et al., 2011	49 non diabetic adults	TTE vs. CMR	TTE of EPI and PERI thickness vs. CMR	PLAX and PSAX, end-diastole, mean of 3 cycles	1.5-T CMR T1-weighted fast spin-echo sequences	Good agreement between TTE and CMR for total cardiac fat; PERI (not EPI) strongly correlated with visceral fat, metabolic syndrome parameters, and 10-year CHD risk; PERI superior cardiometabolic marker.	[22]
Chao et al., 2013	283 patients, 227 with paroxysmal AF and 56 with non paroxysmal AF	TTE vs. cCT	TTE EAT thickness vs. CT volume	PLAX and PSAX end systole		There is significant correlation between TTE and CT values (*r*=0.852, *p*<0.0001)	[65]
Kim et al., 2017	2,276 asymptomatic adults	TTE vs. cCT	TTE EAT thickness vs. cCT volume	PLAX and PSAX end systole	64 slice MDCT	Moderate correlation between TTE-EAT and CT-EAT volume (*r*=0.37; weak agreement k=0.146); both independently associated with coronary artery calcification, with slightly stronger association for TTE-EAT.	[34]
Nerlekar et al., 2018	106 patients undergoing cCT for suspected CAD.	TTE vs. cCT	TTE EAT thickness vs. cCT EAT measurements	PLAX view	Non-contrast cardiac CT	Poor correlation between TTE and CT-derived EAT measurements (*r* = 0.29, *p* = 0.002)	[81]
Parisi et al., 2020	1061 patients undergoing TTE. 70 underwent both TTE and CMR	TTE vs. CMR	TTE EAT thickness vs. CMR thickness and volume	PLAX view; common views and Rindfleisch fold; 3 cardiac cycles; end-systole	1.5 T CMR; short-axis cine SSFP; EAT volume obtained by summation of traced areas (slice thickness + gap: 8 + 2 mm)	Significant correlation between TTE EAT thickness and CMR EAT volume (*r* = 0.61, *p* < 0.0001); stronger at Rindfleisch fold (*r* = 0.71, *p* < 0.001). Proposed cut-off: 10 mm	[56]
Van Woerden et al., 2022	117 patients with HFpEF	TTE vs. CMR	TTE EAT thickness vs. CMR EAT volume	PLAX and PSAX views; mean of repeated measurements; end-systole and end-diastole	1.5 T CMR; volumetric EAT quantification using modified Simpson’s rule	Moderate correlation between TTE and CMR EAT; stronger agreement with experienced observers (*r* = 0.62 vs. *r* = 0.33)	[79]

*Abbreviations: *cCT *cardiac computed tomography, *CMR* cardiac magnetic resonance, *EAT *epicardial adipose tissue, *HFpEF *heart failure with preserved ejection fraction, *PLAX* parasternal long axis view, *PSAX *parasternal short axis view, *TTE* trans-thoracic echocardiography

Disease-specific clinical studies evaluate the association between TTE-derived EAT thickness. These studies are synthesized qualitatively due to variability in measurement protocols and reported outcomes **(**Table 1**)**.

Validation studies assess the relationship between one-dimensional TTE-derived EAT thickness and volumetric EAT quantification by cross-sectional imaging. Four studies compared TTE-derived EAT thickness with CMR-derived EAT volume, whereas three studies evaluated the association between TTE and cCT-derived EAT measurements. Quantitative synthesis was restricted to CMR studies because the available cCT studies were characterized by substantial methodological heterogeneity. Specifically, some studies compared TTE EAT thickness with cCT-derived linear thickness measurements, whereas others compared TTE thickness with cCT-derived volumetric EAT quantification. In addition, differences in image acquisition, EAT segmentation protocols, anatomical reference points, and volume calculation methods limited direct comparability across studies. Consequently, pooling cCT-based studies was deemed inappropriate and these studies were analysed qualitatively.

Across studies, TTE-based EAT assessment is performed using one-dimensional thickness measurements obtained from PLAX or PSAX or other standardized imaging planes. Reference imaging modalities (CMR and cCT) quantify EAT using volumetric approaches.

A subset of included studies additionally reports longitudinal CV outcomes and is therefore eligible for inclusion in the exploratory prognostic meta-analysis **(**Table 4**)**.

### Association Between TTE- EAT Thickness and CMR/cCT-derived EAT

All included studies report a positive association between TTE- EAT thickness and EAT volume measured by CMR. Individual correlation coefficients range from *r* = 0.61 to *r* = 0.905, indicating a moderate-to-strong relationship across studies.

**Fig. 3:**
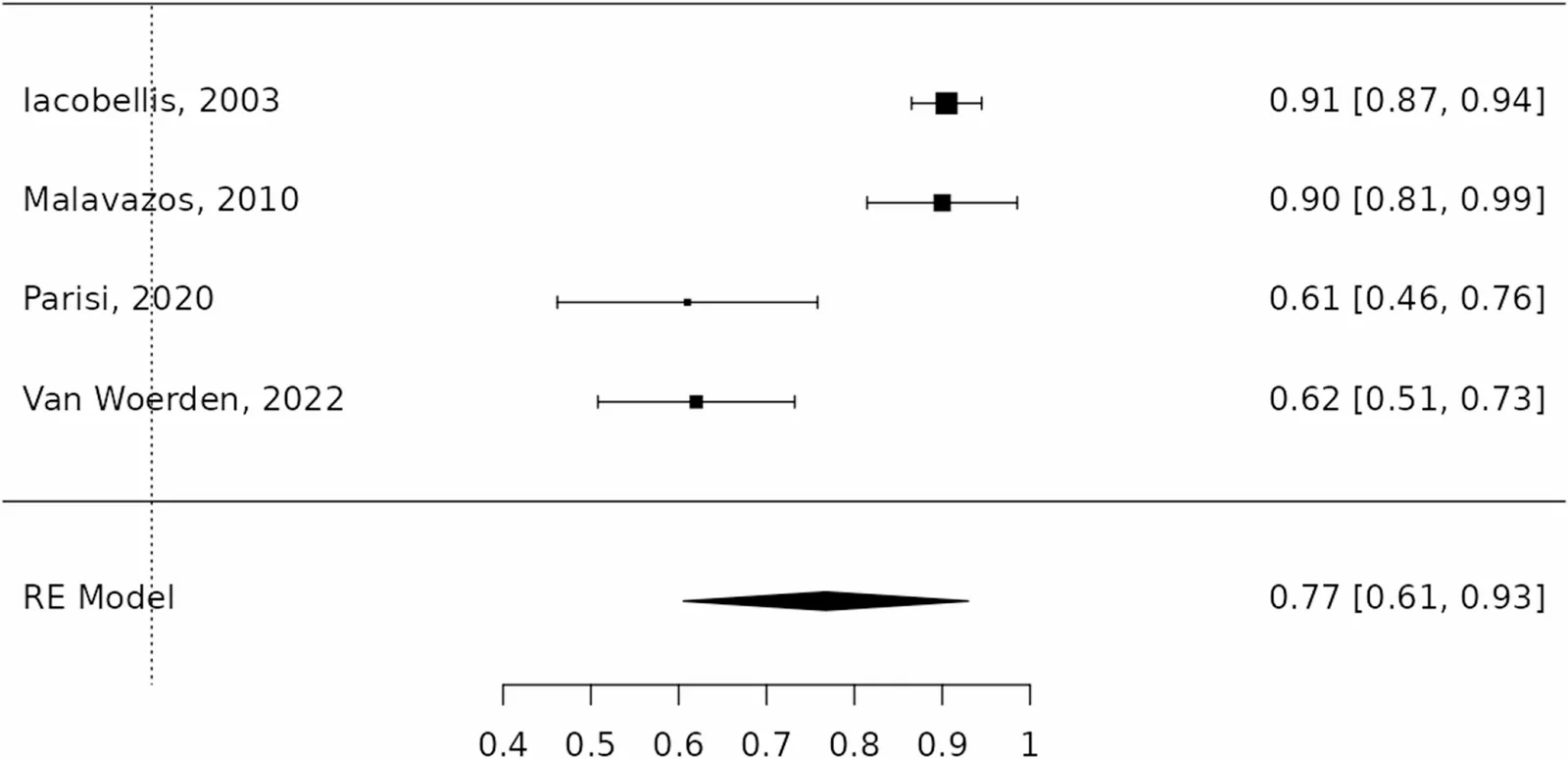
Meta-analytic results of the association between TTE- EAT thickness and CMR-derived EAT

Quantitative synthesis is performed pooling correlation coefficients using a random-effects model. The pooled analysis demonstrates a significant association between TTE- EAT thickness and CMR-EAT measurements, with a pooled correlation coefficient of *r* = 0.768 (95% confidence interval 0.650–0.930 *p* < 0.01) **(Fig. **3**)**.

### Exploratory Prognostic Analysis of Echocardiographic EAT Thickness

The main characteristics of the included prognostic studies are summarized in Table 4. Studies identified some time-to-event outcomes as HRs are pooled using a random-effects model and the generic inverse-variance method. Effect estimates are log-transformed and presented on the logarithmic scale.

**Fig. 4:**
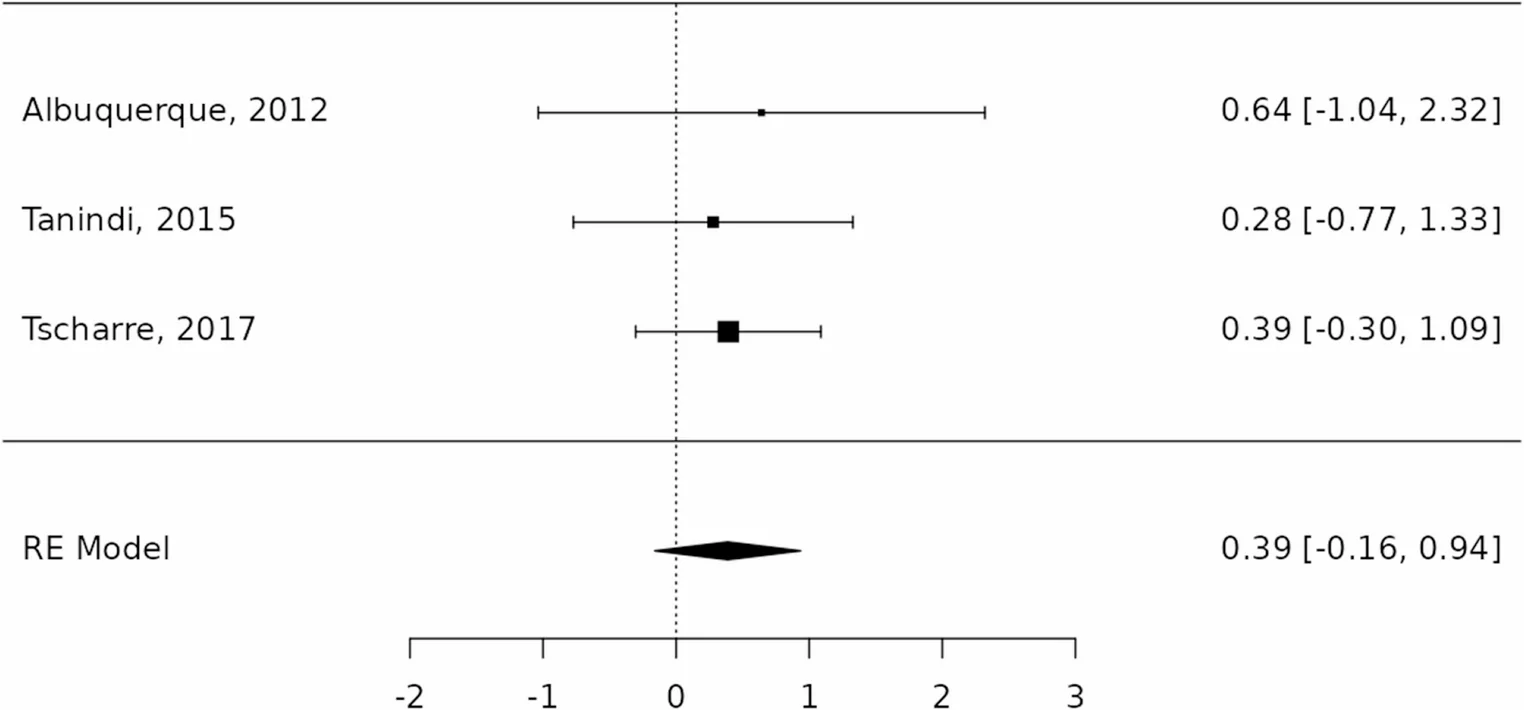
Forest plot of the random-effects exploratory meta-analysis assessing the association between increased TTE- EAT thickness and major adverse CV events, including only studies reporting hazard ratios. Effect estimates are shown on the logarithmic scale (pooled HR 1.50, 95% CI 0.67–3.37; *p* = 0.326)

In the primary exploratory analysis, increased TTE-derived EAT thickness showed a directional association with a higher risk of MACE, although statistical significance was not reached (pooled HR 1.50, 95% CI 0.67–3.37; *p* = 0.326) **(**Fig. 4**)**. The pooled correlation coefficient was 0.80 (95% CI 0.57–0.91; I² = 93%). The corresponding 95% prediction interval ranged from − 0.76 to 0.997, indicating substantial variability in the correlation that may be observed in future studies.

**Fig. 5:**
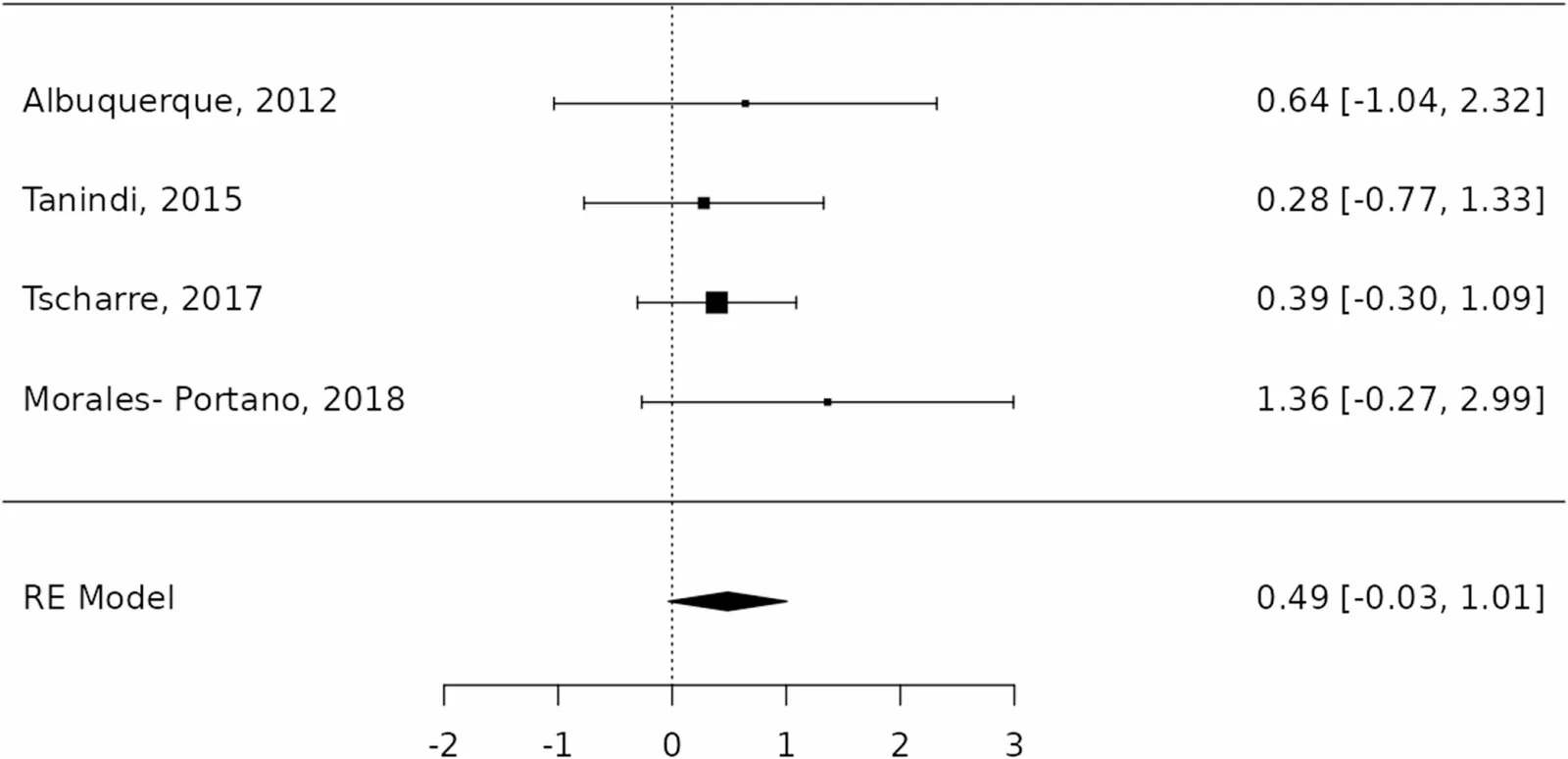
Sensitivity analysis including one study reporting relative risks, treated as an approximation of hazard ratios. Effect estimates are shown on the logarithmic scale HR 1.81 (95% CI 0.88–3.74), *p* = 0.108)

In the sensitivity analysis including one additional study reporting relative risks, the overall direction of the association remained consistent, although pooled estimates remained statistically non-significant, corresponding to a pooled hazard ratio of 1.81 (pooled HR 1.81 (95% CI 0.88–3.74), *p* = 0.108) **(**Fig. 5**).** Given the limited number of studies and the exploratory nature of this analysis, these findings should be interpreted with caution.

### Heterogeneity

**Fig. 6:**
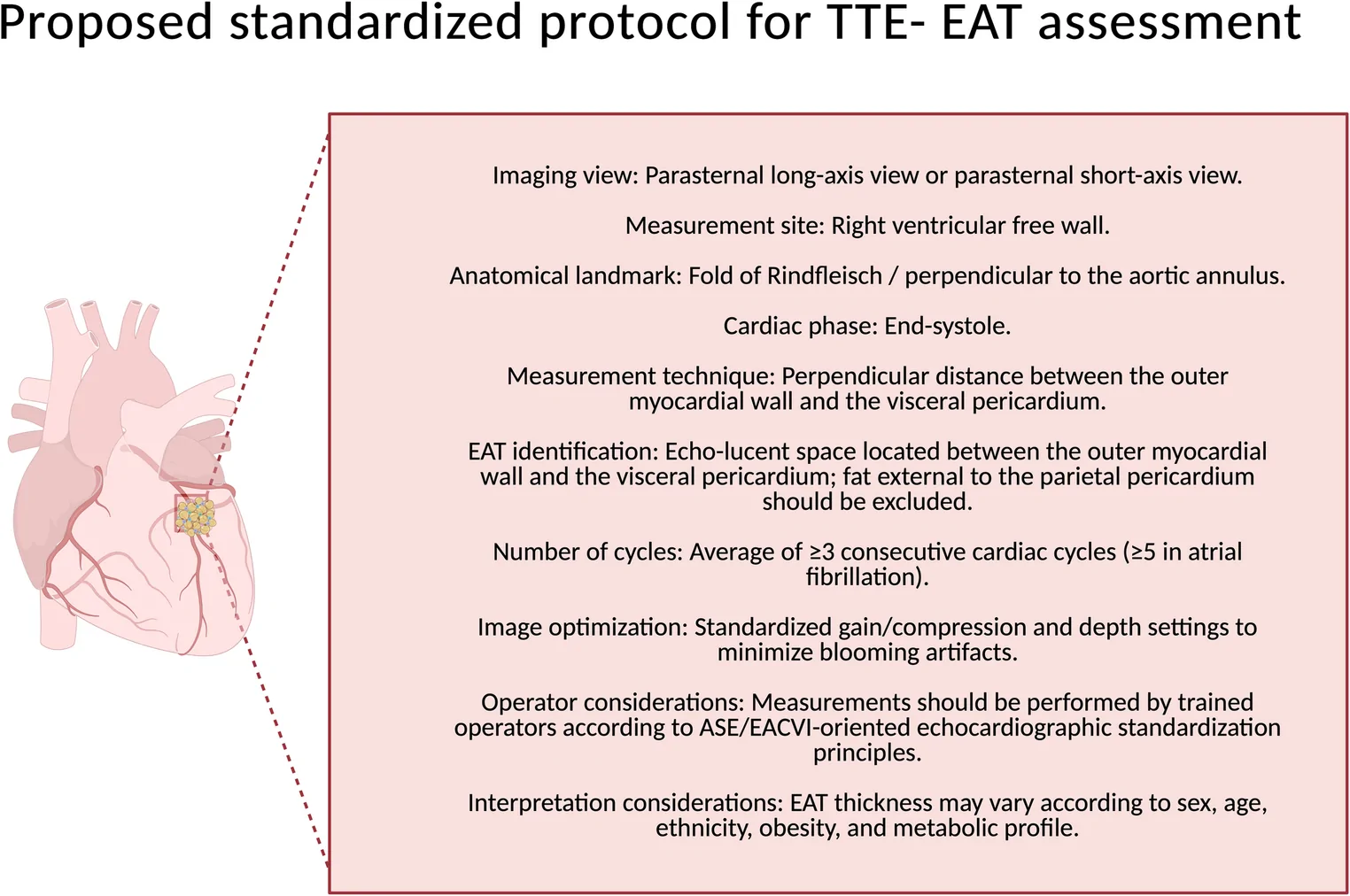
Proposed standardized protocol for transthoracic echocardiographic assessment of epicardial adipose tissue. The figure summarizes a pragmatic stepwise approach for TTE-derived EAT thickness assessment based on the most reproducible methodologies reported in the literature. Recommended acquisition includes measurement at end-systole in the parasternal long-axis view over the right ventricular free wall, using the aortic annulus as an anatomical reference and averaging at least three cardiac cycles. Technical optimization should include appropriate gain and compression settings and careful differentiation between epicardial fat, pericardial fat, and pericardial effusion. The protocol also emphasizes systematic reporting of imaging view, cardiac phase, and measurement methodology in order to reduce operator dependency and improve inter-study comparability. Prospective validation studies are warranted before clinical implementation

Between-study heterogeneity is assessed using the I² statistic and was high (I² = 92.95%), indicating substantial variability among studies. Given the small number of included studies and the substantial heterogeneity in TTE acquisition protocols and reference imaging methodologies, no additional subgroup analyses are performed beyond the predefined sensitivity analysis (Fig. 6).

The methodological quality assessment using the QUADAS-2 tool revealed an overall low risk of bias regarding patient selection and the reference standard. However, significant concerns emerged in the Index Test (TTE) domain. This is primarily linked to the high operator-dependency and the variability in acquisition protocols, as highlighted by the discrepancy in correlation strength between expert and non-expert sonographers. Furthermore, the lack of standardized anatomical landmarks across studies contributes to the observed methodological heterogeneity **(**Table 2**).**

Between-study heterogeneity in the exploratory prognostic meta-analysis is low (I² = 0%). However, given the small number of included studies, the ability to detect true heterogeneity is limited.

Qualitative stratification of the included studies suggests that methodological heterogeneity was largely driven by differences in echocardiographic acquisition protocols. Studies using end-systolic measurements in the parasternal long-axis view tended to report stronger correlations with CMR-derived EAT volume compared with studies using mixed acquisition approaches or end-diastolic measurements. Similarly, greater agreement between TTE and CMR-derived EAT was observed in studies performed by experienced operators, supporting the influence of operator expertise on measurement reproducibility.

### Qualitative Synthesis of Methodological Variability

Considerable heterogeneity is observed in TTE measurement techniques across studies, including differences in imaging views. Despite these methodological differences, the direction of association between TTE- derived EAT thickness and cross-sectional imaging–derived EAT is consistent across all studies. Similarly, in studies reporting longitudinal CV outcomes, increased TTE-derived EAT thickness is consistently associated with a higher risk of adverse events, although the magnitude of effect varied across heterogeneous patient populations and outcome definitions.

### Summary of Main Findings

Overall, the findings of this systematic review and meta-analysis demonstrate that TTE–derived EAT thickness shows a significant and consistent association with volumetric EAT quantification obtained by CMR or cCT. The pooled correlation indicates a moderate-to-strong relationship, although the magnitude of the association is influenced by methodological variability across studies.

Beyond imaging validation, the clinical fields of application of echocardiographic EAT detection appear to be broad, particularly in high-risk populations. Indeed, a substantial body of literature has investigated the role of TTE-derived EAT thickness across diverse CV conditions, reflecting growing interest in its potential utility for cardiometabolic risk assessment and disease phenotyping. In this context, the systematic synthesis of disease-specific clinical studies supports a consistent association between increased TTE-derived EAT thickness and major CV phenotypes.

Exploratory prognostic analyses further suggest a directional association between increased TTE-derived EAT thickness and adverse CV outcomes. However, these findings should be interpreted cautiously given the limited number of longitudinal studies and the presence of methodological heterogeneity.

## Discussion

The exploratory prognostic findings of the present study should be interpreted cautiously. Although the pooled estimates showed a consistent directional association between increased TTE-derived EAT thickness and adverse CV outcomes, the limited number of available studies, wide confidence intervals, and methodological heterogeneity substantially limit the strength of inference.

Accordingly, these findings should be considered hypothesis-generating and primarily supportive of the rationale for future prospective multicenter studies specifically designed to evaluate the prognostic role of TTE-derived EAT assessment.

### Disease-specific Clinical Phenotypes: CAD, AF, and HFpEF

Beyond imaging validation, the present systematic synthesis highlights a reliable relationship between increased TTE-derived EAT thickness and distinct CV phenotypes, namely CAD, AF, and HFpEF [60–76].

In CAD, multiple studies demonstrate that greater EAT thickness is associated not only with the presence of angiographically confirmed coronary disease but also with its anatomical severity, including higher Gensini and SYNTAX scores [68–76]. In acute coronary settings, increased EAT has been linked to adverse procedural outcomes such as no-reflow and impaired coronary perfusion [69, 70]. Furthermore, several investigations report that EAT thickness provides incremental prognostic information beyond traditional risk markers [58, 64] and only one research article is against this hypothesis [72]. These findings support the concept of EAT as a local pro-inflammatory and pro-atherogenic depot capable of modulating coronary plaque burden and vulnerability [13].

In AF, EAT appears to contribute to structural and electrical remodeling of the atria [13, 14]. Increased EAT thickness is associated with AF chronicity and recurrence after catheter ablation [66], as well as with postoperative AF in cardiac surgery populations [65]. The mechanistic link may involve EAT–mediated inflammatory signaling and local fibrotic remodeling at the atrial–epicardial interface, consistent with prior pathophysiological models [14]. These data suggest that TTE-derived EAT thickness may reflect an arrhythmogenic substrate rather than merely a marker of systemic adiposity [13, 14].

In HFpEF, the association between EAT and adverse hemodynamic and functional profiles appears particularly relevant [60–62]. Increased EAT thickness has been associated with diastolic dysfunction [62] and with a more severe HFpEF phenotype characterized by higher filling pressures, worse pulmonary vascular coupling, and reduced exercise capacity [60, 61]. These observations support the hypothesis that EAT may contribute not only through inflammatory pathways but also via mechanical constraint and ventricular interdependence, potentially exacerbating the restrictive physiology typical of HFpEF [15–17].

Taken together, these disease-specific findings reinforce the clinical relevance of TTE-derived EAT thickness across diverse CV conditions. While causality cannot be inferred from predominantly observational data, the consistency of associations across CAD, AF, and HFpEF suggests that EAT may represent a shared pathophysiological mediator linking cardiometabolic dysregulation to structural and functional cardiac remodeling [15–17].

In order to translate these clinical associations into practical implementation, it is essential to determine whether TTE-derived EAT thickness reliably reflects total EAT burden as quantified by cross-sectional imaging.

Indeed, the main finding of our work is that TTE- derived EAT thickness shows a significant correlation with EAT quantified by CMR [32, 54, 56, 77, 78].

These results suggest that simple linear TTE measurements describe, at least in part, the overall EAT burden, while also highlighting the inherent limitations of one-dimensional assessment for a three-dimensional and spatially heterogeneous tissue.

Nonetheless, the interpretation of these findings requires careful consideration of the intrinsic limitations of TTE- EAT assessment. Unlike CMR and cCT, which provide three-dimensional volumetric quantification, TTE relies on one-dimensional linear measurements obtained from selected anatomical planes. Consequently, TTE-derived EAT thickness cannot fully capture the complex spatial heterogeneity and regional distribution of EAT across the cardiac surface.

This limitation is particularly relevant given the heterogeneous anatomical distribution of EAT, which varies substantially across atrioventricular grooves, ventricular free walls, and pericoronary regions. Therefore, one-dimensional TTE measurements should be interpreted as simplified surrogates of total EAT burden rather than direct volumetric equivalents.

However, this limitation should be balanced against the substantial practical advantages of TTE. Unlike cross-sectional imaging modalities, TTE is widely available, inexpensive, radiation-free, and easily repeatable, making it particularly suitable for large-scale cardiometabolic screening and longitudinal follow-up. Accordingly, TTE should not be interpreted as a volumetric substitute for CMR or cCT, but rather as a pragmatic phenotyping tool capable of identifying high-risk cardiometabolic profiles in routine clinical practice. The lack of a universally accepted standard regarding the cardiac phase at which measurements should be performed remain an important issue. While several studies describe EAT thickness at end-systole, following the original methodological descriptions, others perform measurements in end-diastole, thereby contributing to methodological heterogeneity across investigations **(**Table 1**-** Table 3**).**

**Table 3:**
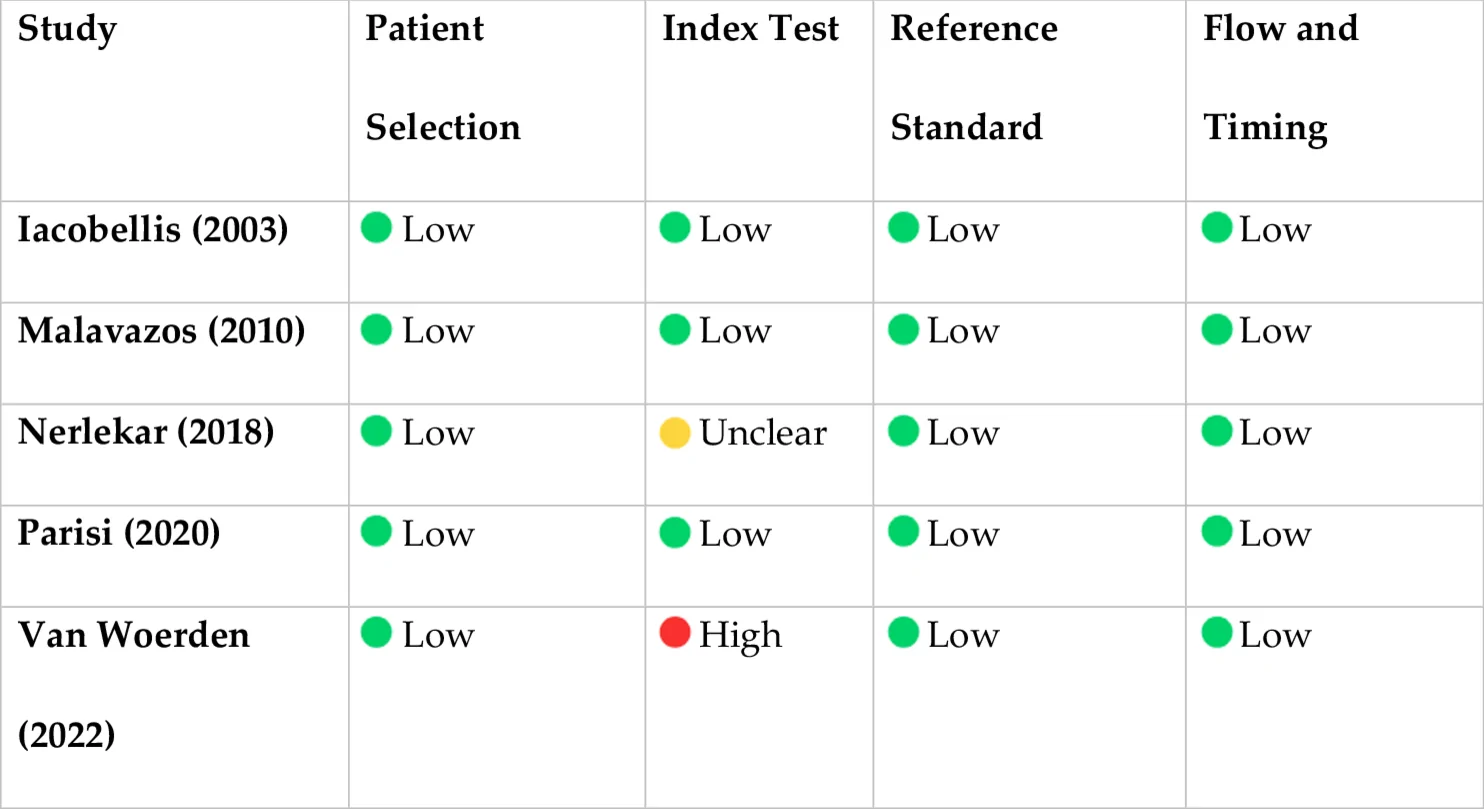
QUADAS-2 quality assessment of included studies

Importantly, methodological heterogeneity likely represents one of the principal determinants of the high between-study variability observed in the pooled analysis. Studies adopting end-systolic acquisition in the PLAX view, particularly at the level of the right ventricular free wall referenced to the aortic annulus, generally demonstrated better reproducibility and stronger correlations with CMR-derived EAT volume. This observation is consistent with previous methodological investigations suggesting that end-systolic measurements may provide improved delineation of epicardial fat due to myocardial contraction and enhanced separation from adjacent pericardial structures.

In addition, operator expertise appears to substantially influence measurement reliability. As highlighted by van Woerden et al. [79] correlations between TTE and CMR-derived EAT measurements were significantly stronger when acquisitions were performed by experienced observers, emphasizing the need for dedicated training and standardized acquisition protocols before broad clinical implementation (Table 4).

**Table 4:** Prognostic studies evaluating the association between TTE–derived EAT and CV outcomes

Author, year	Study population	*N*	Echocardiographic assessment of EAT	Follow-up	EAT cut-off	Outcome	Effect estimate
Albuquerque et al., 2012 [59]	Patients with CAD	194	PLAX view; 3 cardiac cycles	26 months	7 mm	MACE	HR 1.90 (95% CI 0.40–8.30), *p* = 0.039
Tanindi et al., 2015 [62]	Patients with CAD	200	Parasternal long-axis view; end-systole; mean of 3 cycles	26 months	7 mm	MACE / CV death	HR 1.32 (95% CI 0.75–2.31), *p* = 0.33
Chu et al., 2016 [67]	Patients with AF	190	PLAX; end-diastole; mean of 3 cycles	29 months	Continuous (per 1-mm increase)	Composite CV events	HR 1.22 (95% CI 1.10–1.37), *p* < 0.001
Tscharre et al., 2017 [74]	Patients with ACS undergoing PCI	438	PLAX; end-systole; mean of 3 cycles	3 years	Not specified	MACE	HR 1.48 (95% CI 1.19–1.95), *p* = 0.006
Morales-Portano et al., 2018 [64]	Patients with CAD	107	Two-dimensional and M-mode TTE; end-systole; mean of 10 cycles	16 months	4.6 mm	MACE	RR 3.91 (95% CI 1.01–15.08), *p* = 0.04

*Abbreviations: *ACS* acute coronary syndromes, *AF* atrial fibrillation, *CAD* coronary artery disease, *CV* cardiovascular, *EAT* epicardial adipose tissue, *HR* hazard ratio, *MACE* major adverse cardiovascular events, *PLAX* parasternal long axis view, *TTE* trans-thoracic echocardiography

In addition, there is no consensus regarding the optimal TTE projection for EAT assessment, with different studies adopting distinct imaging planes and anatomical landmarks. Furthermore, we provide an additional illustrative figure obtained in a single patient, showing EAT thickness measured using the different TTE projections (Fig. 1**).** This figure was developed inspired by the comprehensive TTE overview proposed by Ana Duzé et al. [58], with the aim of visually summarizing the acquisition approaches reported in the literature. In this example, measurements obtained from the various views appear largely overlapping, suggesting that, when properly acquired, different projections may yield comparable estimates of EAT thickness. Nevertheless, the PLAX view remains the most frequently adopted projection in the available literature **(**Fig. 1**).**

The present study should be regarded as a starting point rather than a definitive validation of TTE- EAT assessment. From a clinical perspective, it is neither realistic nor appropriate to perform cCT—even in the absence of contrast but still involving ionizing radiation—or CMR imaging for preventive risk stratification in asymptomatic individuals [69, 72]. In this context, the availability of a simple, time-sparing, and widely accessible tool such as TTE represents a major potential advantage [46].

This clinical need provides the rationale for conducting an exploratory meta-analysis focused on MACE and their association with TTE-derived EAT thickness. The results of this analysis are directionally consistent with those of the individual studies, suggesting that increased TTE-measured EAT e is associated with a higher risk of MACE [64, 67, 69, 72, 80]. No substantial statistical heterogeneity was detected; however, given the limited number of included studies and the exploratory nature of the analysis, this association does not reach definitive statistical significance and should be interpreted with caution.

Within this context, if adequately standardized, TTE- EAT assessment could represent a pragmatic tool for broader and more frequent evaluation of cardiometabolic risk in routine clinical practice, extending beyond selected high-risk populations [46].

The exploratory prognostic signal observed in the present analysis supports the rationale for future prospective studies specifically designed to evaluate the role of TTE-derived EAT thickness in CV risk stratification. Moreover, TTE would be uniquely suited for longitudinal monitoring, allowing assessment of changes in EAT burden over time and evaluation of treatment response to emerging pharmacological therapies targeting adipose tissue, such as glucagon-like peptide-1 receptor agonists and dual incretin agonists (e.g., tirzepatide) [73].

Nevertheless, widespread clinical implementation of TTE- EAT measurement requires not only methodological standardization but also operator training. Experience is essential as suggested by van Woerden et al., who demonstrate a stronger correlation between TTE and CMR-derived EAT measurements when assessments are performed by more expert operators [77].

In addition, alternative strategies may further enhance the clinical relevance of TTE-EAT assessment [58]. As suggested by Ana Đuzel Čokljat and colleagues, normalization of EAT thickness to waist circumference may provide a metric more closely associated with CV risk than absolute EAT values alone [58].

Furthermore, given the dynamic and metabolically active nature of EAT, longitudinal changes in EAT (ΔEAT) may be more informative than single absolute measurements, particularly in the context of preventive interventions and therapeutic monitoring. Moreover, future diagnostic guidelines should explore the systematic use of the Rindfleisch fold as the primary TTE landmark for EAT quantification [56].

Taken together, these considerations support the role of TTE as a pragmatic tool for EAT assessment, while underscoring the need for standardization, operator training, and longitudinal validation before its full integration into preventive CV strategies.

### Comparison with Cardiac Computed Tomography

Although cCT is widely regarded as a robust modality for EAT quantification, the number of studies directly comparing TTE-derived EAT thickness with cCT-derived EAT remains limited [32]. Moreover, available studies have reported inconsistent findings, ranging from moderate-to-strong correlations to relatively weak associations.

In particular, Nerlekar et al. reported a poor correlation between TTE and cCT-derived EAT measurements (*r* = 0.29), highlighting the potential limitations of one-dimensional TTE assessment when compared with volumetric cCT quantification [78]However, given the limited number of available studies and the substantial methodological variability across imaging protocols, definitive conclusions regarding TTE–cCT agreement cannot currently be established.

Consequently, the present quantitative synthesis mainly reflects the relationship between TTE-derived EAT thickness and CMR-derived EAT volume, whereas further dedicated comparative studies with cCT are warranted.

### Clinical Implications

From a clinical standpoint, the results of this meta-analysis support a complementary, rather than competitive, role of TTE within a multimodality imaging framework for EAT assessment. TTE-EAT thickness may provide a practical first-line estimate of EAT and help identify individuals who could benefit from more detailed volumetric assessment using CMR or cCT, including evaluation of PCAT.

In this context, TTE may be particularly useful in clinical scenarios requiring repeated assessments over time, where accessibility, feasibility and absence of radiation exposure are key considerations. Moreover, this approach may provide a useful tool for assessing the impact of dietary interventions on CV risk modulation. Given the dynamic nature of EAT in response to lifestyle modification, TTE-EAT could represent a pragmatic surrogate marker to monitor longitudinal changes in visceral cardiac adiposity, especially in real-world and preventive settings. However, at present caution is warranted when using TTE-EAT measurements for precise risk stratification or inter-individual comparisons. Nonetheless, the exploratory prognostic signal observed in the present analysis suggests that the clinical relevance of TTE-measured EAT thickness may extend beyond mere anatomical quantification. Unlike the diffuse fat depots in the body, EAT is a transcriptionally active visceral tissue directly apposed to the myocardium and sharing the same microcirculation [2]. In this context, TTE- EAT thickness be representative of the cardiac “inflammo-metabolic” milieu, reflecting local secretion of pro-inflammatory cytokines (e.g., interleukin-6, tumor necrosis factor-α, and monocyte chemoattractant protein-1) that may promote myocardial fibrosis and coronary plaque instability through paracrine and vasocrine signaling [2].

While CMR and cCT offer superior volumetric precision, TTE provides a simple and readily available assessment of EAT thickness, which may serve as a surrogate marker of overall EAT burden, although it does not directly assess the metabolic or inflammatory activity ofEAT. Increased EAT thickness at TTE has been associated with higher levels of systemic inflammatory markers and impaired adiponectin signalling, supporting its potential pathophysiological relevance [3].

Finally, the feasibility of serial TTE assessments enables longitudinal monitoring of EAT thickness as a dynamic biomarker of therapeutic response. Taken together, these considerations indicate that TTE-EAT assessment should not be viewed solely as a simplified surrogate for volumetric imaging, but rather as a pragmatic bedside tool for cardiometabolic phenotyping—while acknowledging that prospective, standardized studies are required before clinical implementation. Building on these considerations, we propose a pragmatic, stepwise TTE algorithm for EAT assessment aimed at improving methodological consistency and facilitating clinical translation of TTE-derived EAT measurements **(**Fig. 6**).**

Within a multimodality imaging framework, TTE may therefore represent an effective first-line approach for EAT assessment, reserving CMR or cCT for selected cases requiring precise volumetric characterization or detailed evaluation of PCAT.

Furthermore, biological variability related to sex, age, and ethnicity should also be considered when interpreting TTE-derived EAT measurements. Previous studies suggest that EAT distribution and cardiometabolic significance may differ substantially across populations, supporting the future development of sex- and ethnicity-adjusted reference ranges rather than a single universal threshold.

### Emerging Technologies and Future Directions

Recent technological developments may substantially improve the clinical applicability and reproducibility of EAT assessment across imaging modalities. In particular, artificial intelligence (AI)-based approaches for automated EAT segmentation and quantification have shown promising results in both TTE and CMR [82].

Deep learning algorithms applied to TTE videos may allow semi-automated or fully automated EAT thickness assessment, potentially reducing operator dependency and improving inter-observer reproducibility [83]. Similarly, automated CMR-based segmentation tools have demonstrated increasing accuracy for volumetric EAT quantification while substantially reducing post-processing time [82].

Beyond conventional anatomical measurements, emerging imaging approaches are increasingly focusing on the functional and inflammatory characterization of EAT. In cCT imaging, EAT attenuation and pericoronary FAI analysis provide indirect information regarding local coronary inflammation and adipose tissue remodelling [84]. Likewise, radiomics-based tissue heterogeneity analysis and texture characterization may offer additional insights into the biological activity of EAT beyond simple thickness or volume measurements.

Future developments integrating AI-assisted acquisition, automated quantification, and functional tissue characterization may facilitate broader standardization of EAT assessment and support its incorporation into routine cardiometabolic risk stratification frameworks.

Future research should prioritize large prospective multicenter studies using standardized TTE acquisition protocols and harmonized outcome definitions [82–84]. In particular, longitudinal assessment of dynamic EAT changes (ΔEAT) may provide clinically relevant information regarding treatment response and cardiometabolic risk modification. AI-assisted TTE acquisition and automated EAT quantification may further improve reproducibility and facilitate broader clinical implementation [82–84].

### Limitations

Several limitations have to be acknowledged. First, the number of included studies in meta-analyses is small, limiting the ability to perform subgroup or sensitivity analyses and precluding formal assessment of publication bias. Second, methodological heterogeneity across studies, particularly in TTE acquisition techniques, may have influenced the pooled estimates. Third, the meta-analysis focuses exclusively on correlation metrics, and agreement analyses between modalities could not be quantitatively synthesized. Finally, the limited availability of studies directly comparing TTE with cCT restricts the generalizability of the findings across imaging modalities.

With regard to the exploratory prognostic analysis, additional limitations should be acknowledged. The number of studies reporting longitudinal outcomes is very limited, resulting in low statistical power to detect true between-study heterogeneity and precluding a formal risk-of-bias assessment specific to prognostic studies. Therefore, although no substantial statistical heterogeneity is observed, these findings should be interpreted cautiously and considered hypothesis-generating rather than definitive.

In addition, the limited number of studies directly comparing TTE with cCT-derived EAT measurements restricts the ability to generalize findings across imaging modalities.

Additional limitations specifically apply to the exploratory prognostic meta-analysis. The number of studies reporting longitudinal outcomes was small, limiting statistical power and precluding formal publication bias assessment or robust subgroup analyses. Furthermore, outcome definitions, follow-up durations, and EAT cut-off values differed substantially across studies. The inclusion of both HR- and RR-based effect estimates, although limited to sensitivity analyses, may also have contributed to methodological variability.

## Conclusions

In conclusion, TTE -EAT thickness had a wide applicability and is significantly correlated with CMR-derived EAT volume, supporting its potential role as a simple and widely accessible marker of EAT.

By integrating data from multiple studies and addressing sources of methodological heterogeneity, this meta-analysis extends prior work and offers a more robust framework for interpreting TTE-EAT measurements within a multimodality imaging context. Exploratory prognostic analyses further suggest a potential association between increased TTE-EAT thickness and adverse CV outcomes. Standardization of acquisition protocols and further prospective validation are required before TTE-EAT measurement can be fully integrated into routine clinical risk stratification.

## Key References

- Fang, W.; Xie, S.; Deng, W. Epicardial Adipose Tissue: A Potential Therapeutic Target for Cardiovascular Diseases. J. Cardiovasc. Transl. Res. 2024, 17, 322–333, https://doi.org/10.1007/s12265-023-10442-1.
  - ○  This recent review summarizes emerging evidence on the pathophysiological role of epicardial adipose tissue and its potential as a therapeutic target in cardiovascular disease.
- Iacobellis, G. Epicardial Adipose Tissue in Contemporary Cardiology. Nat. Rev. Cardiol. 2022, 19, 593–606, https://doi.org/10.1038/s41569-022-00679-9.
  - ○  This comprehensive review provides a state-of-the-art overview of epicardial adipose tissue as a metabolically active organ, highlighting its central role in cardiovascular risk and its relevance as a clinical imaging biomarker.
- Napoli, G.; Pergola, V.; Basile, P.; De Feo, D.; Bertrandino, F.; Baggiano, A.; Mushtaq, S.; Fusini, L.; Fazzari, F.; Carrabba, N.; et al. Epicardial and Pericoronary Adipose Tissue, Coronary Inflammation, and Acute Coronary Syndromes. J. Clin. Med. 2023, 12, 7212, https://doi.org/10.3390/jcm12237212.
  - ○  This study highlights the link between epicardial and pericoronary adipose tissue and coronary inflammation, supporting the concept of EAT as a local driver of atherosclerosis.
- Janssen-Telders, C.; Eringa, E.C.; de Groot, J.R.; de Man, F.S.; Handoko, M.L. The Role of Epicardial Adipose Tissue Remodelling in Heart Failure with Preserved Ejection Fraction. Cardiovasc. Res. 2025, 121, 860–870, https://doi.org/10.1093/cvr/cvaf056.
  - ○  This recent work provides mechanistic insights into the role of epicardial adipose tissue in HFpEF, emphasizing its contribution to myocardial stiffness and cardiometabolic dysfunction.
- Iacobellis, G.; Assael, F.; Ribaudo, M.C.; Zappaterreno, A.; Alessi, G.; Di Mario, U.; Leonetti, F. Epicardial Fat from Echocardiography: A New Method for Visceral Adipose Tissue Prediction. Obes. Res. 2003, 11, 304–310, https://doi.org/10.1038/oby.2003.45.
  - ○  This seminal study first introduced and validated echocardiographic measurement of epicardial adipose tissue, establishing the methodological foundation for subsequent research and clinical applications.

## Data Availability

No datasets were generated or analysed during the current study.

## Abbreviations

AF: atrial fibrillation
AV: atrio-ventricular
EAT: epicardial adipose tissue
BMI: body mass index
CAD: coronary artery disease
cCT: cardiac computed tomography
CCTA: contrast-enhanced coronary computed tomography angiography
CMR: cardiac magnetic resonance
CV: cardiovascular
CT: computed tomography
DM2: type 2 diabetes mellitus
FAI: fat attenuation index
HFpEF: heart failure with preserved ejection fraction
HU: Hounsfield unit
IL-8: interleukin-8
HFpEF: heart failure with preserved ejection fraction
LAD: left anterior descending artery
MACE: major adverse cardiovascular events
NCCT: non contrast cardiac computed tomography
PAT: pericardial adipose tissue
PCAT: pericoronary adipose tissue
PLAX: parasternal long axis
PSAX: parasternal short axis
ROC: receiver operating curve
SSFP: steady state free precession
TTE: trans-thoracic echocardiography
VAT: visceral adipose tissue
