# Correction to: The Role of Echocardiography in the Assessment of Epicardial Adipose Tissue: A Systematic Review and Meta-analysis

**DOI:** 10.1007/s13679-026-00769-6

**Published:** 2026-10-10

**Authors:** Barbara Pala, Mariagrazia Piscione, Francesco Cribari, Serena De Mitri, Paola Gualtieri, Antonino De Lorenzo, Luigi Barrea, Marco Alfonso Perrone, Gianluca Iacobellis, Laura Di Renzo

**Affiliations:** 1 PhD School of Applied Medical-surgical Sciences, Tor Vergata University of Rome, Via Montpellier, 1, Rome, 00133 Italy; 2 UOC Cardiologia Ospedale IDI- IRCCS, Via Monti di Creta, 104, Rome, 00167 Italy; 3 ASL 2 Abruzzo, Santissima Annunziata Hospital, Via dei Vestiti, Chieti, 66100 Italy; 4 Section of Food Science, Clinical Nutrition and Pharmaceutical Sciences, Department of Biomedicine and Prevention, Tor Vergata University of Rome, Via Montpellier 1, Rome, 00133 Italy; 5 Dipartimento di Psicologia e Scienze della Salute, Università Telematica Pegaso, Naples, Italy; 6 Division of Cardiology and CardioLab, Department of Clinical Sciences and Translational Medicine, Tor Vergata University of Rome, Via Montpellier,1, Rome, 00133 Italy; 7 Division of Endocrinology, Diabetes and Metabolism, Department of Medicine, Iacobellis Gianluca, University of Miami, Leonard M. Miller School of Medicine, Miami, FL USA

**Correction to: Current Obesity Reports (2026) 15:63**

**https://doi.org/10.1007/s13679-026-00734-3**

In the original version of this article, the names of the authors Antonino De Lorenzo and Gianluca Iacobellis appear to have been incorrectly presented, with their given names and family names reversed.

The correct author details should be:

Given name: Antonino — Family name: De Lorenzo

Given name: Gianluca — Family name: Iacobellis

The original article has been corrected.

